# Allogeneic Hematopoietic Cell Transplantation for Relapsed or Refractory Mantle Cell Lymphoma: Real‐World Outcomes, Late Relapse Patterns, and Clinical Utility in the Chimeric Antigen Receptor T‐Cell Era

**DOI:** 10.1002/jha2.70318

**Published:** 2026-05-26

**Authors:** Enver Aydilek, Paolo Mazzeo, Justin Hasenkamp, Markus Maulhardt, Tobias Tix, Evgenii Shumilov, Gerald Wulf

**Affiliations:** ^1^ Department for Hematology and Medical Oncology University Medical Center Goettingen Goettingen Germany; ^2^ Department of Internal Medicine, Hematology, Oncology, Stem Cell Transplantation and Palliative Medicine, Campus Bielefeld‐Bethel, Protestant Hospital Bethel University Hospital OWL Bielefeld Germany; ^3^ INDIGHO Laboratory Department for Hematology and Medical Oncology University Medical Center Goettingen Goettingen Germany; ^4^ Department of Medicine III—Hematology/Oncology LMU University Hospital LMU Munich Munich Germany; ^5^ Department of Medicine A, Hematology, Oncology and Pneumology, University Hospital Münster Münster Germany

**Keywords:** allogeneic hematopoietic cell transplantation, CAR‐T era, late relapse, mantle cell lymphoma, relapsed or refractory disease

## Abstract

**Background:**

Allogeneic hematopoietic cell transplantation (alloHCT) remains a curative option for relapsed or refractory (r/r) mantle cell lymphoma (MCL), although its role has shifted in the era of Bruton tyrosine kinase inhibition (BTKi) and chimeric antigen receptor T‐cell (CAR‐T) therapy. Real‐world evidence on long‐term outcomes and salvage strategies after relapse is limited.

**Methods:**

We performed a retrospective analysis of 29 patients with r/r MCL who underwent alloHCT between 2001 and 2017 using either higher‐intensity or reduced‐intensity conditioning. Outcomes assessed included overall survival (OS), progression‐free survival (PFS), relapse incidence, and non‐relapse mortality (NRM), focusing on relapse timing and management. Conditioning regimens were compared exploratorily.

**Results:**

With a median follow‐up of 143 months (95% CI: 104–NR), 3‐year OS and PFS were 41% and 34%, respectively. Relapse occurred in 38% of patients, including over one quarter with late (> 12‐month), predominantly localized relapse. Salvage radiotherapy, alone or combined with BTKi, achieved durable disease control in about two‐thirds of patients. Differences between higher‐intensity and reduced‐intensity conditioning were observed but remained exploratory and not statistically conclusive.

**Conclusion:**

In this real‐world cohort, alloHCT provided sustained disease control for a subset of r/r MCL patients, including late, localized relapse amenable to salvage therapy. As alloHCT is now performed less frequently, such datasets are critical for decision‐making in patients after CAR‐T ineligibility or failure, especially where CAR‐T access is limited. These findings support a continued, selective role for alloHCT in the modern treatment landscape and emphasize the need for real‐world evidence to guide patient selection and sequencing in the CAR‐T era.

**Trial Registration:**

The authors have confirmed clinical trial registration is not needed for this submission.

## 1 Introduction

Mantle cell lymphoma (MCL) is a rare, biologically heterogeneous B‐cell non‐Hodgkin lymphoma accounting for approximately 3%–10% of adult lymphomas [1]. Although indolent variants exist, many patients experience an aggressive course characterized by early relapse, therapy resistance, and limited long‐term survival [2]. Adverse molecular and morphologic features such as TP53 mutations, high Ki‐67 proliferation index, and blastoid or pleomorphic histology are associated with inferior outcomes [3, 4].

Over the past decade, the therapeutic landscape of MCL has expanded markedly. Modern frontline regimens combining cytarabine‐based chemo‐immunotherapy with Bruton tyrosine kinase inhibition (BTKi) have improved remission quality and durability [5]. Nevertheless, relapse remains common, and a substantial fraction of patients progress within 24 months (POD24), a strong predictor of poor survival [6]. In the relapsed/refractory (r/r) setting, covalent BTKi such as ibrutinib and acalabrutinib achieve median response durations of 17–19 months [7, 8], while non‐covalent agents like pirtobrutinib show activity after BTKi failure but are not curative [9].

Chimeric antigen receptor T‐cell (CAR‐T) therapy has redefined second‐ or later‐line management. Brexucabtagene autoleucel (brexu‐cel, KTE‐X19) induces overall response rates above 90% in ZUMA‐2 [10], and lisocabtagene maraleucel (liso‐cel) has recently demonstrated comparable efficacy [11]. However, 40%–60% of patients ultimately relapse within 3–5 years of follow‐up, and treatment is not feasible for all due to age, comorbidities, or limited access [12]. Moreover, prolonged cytopenias, immune dysregulation, and infection‐related morbidity highlight that long‐term hematopoietic toxicity after CAR‐T remains incompletely defined [13]. Recent data also indicate a non‐relapse mortality (NRM) exceeding 10% after CAR‐T in MCL [14]. TP53‐altered and highly proliferative MCL appear particularly prone to early progression post‐CAR‐T [15]. Particularly for patients with relapse after CAR‐T, a durable cure remains elusive.

Allogeneic hematopoietic cell transplantation (alloHCT) was historically the only treatment capable of long‐term remission through a graft‐versus‐lymphoma (GvL) effect [16, 17]. Reported 5‐year overall survival (OS) ranges from 30% to 60%, depending on disease status and conditioning intensity [18, 19]. Although its use has declined in the modern era, alloHCT continues to serve selected patients, including those who were ineligible for CAR‐T, those relapsing after CAR‐T, or those with TP53‐mutated disease for whom immune‐mediated tumor control remains a rational curative approach [20, 21].

Although the total number of alloHCT procedures for MCL has decreased, long‐term single‐center datasets remain uniquely valuable for characterizing late and localized relapse patterns, salvageability after recurrence, and durable immune‐mediated disease control—features that cannot be adequately captured in contemporary CAR‐T studies with limited follow‐up [22, 23]. Deeply phenotyped cohorts allow reconstruction of posttransplant relapse management, including radiotherapy, donor lymphocyte infusion (DLI), and BTKi re‐challenge, which remain clinically relevant when alloHCT is considered after CAR‐T ineligibility or failure [21].

The present study provides one of the few real‐world analyses with extended follow‐up, assessing 29 patients with r/r MCL who underwent alloHCT using either higher‐intensity (fludarabine, busulfan, cyclophosphamide; FBC) or reduced‐intensity (fludarabine, cyclophosphamide; FC) conditioning. An exploratory comparison of both conditioning approaches is included. We specifically examine relapse timing, late localized recurrence, and posttransplant salvage approaches including radiotherapy, BTKi re‐challenge, and DLI. These results delineate durable disease control with alloHCT but inform contemporary sequencing in the CAR‐T era, where long‐term outcomes remain largely undefined.

## 2 Methods

We retrospectively analyzed all adult patients with r/r MCL undergoing alloHCT (FBC *n* = 21; FC *n* = 8) in the Department of Hematology and Medical Oncology, University Hospital Goettingen, Germany, from 2001 to 2017. The MCL diagnosis was established by local pathologists according to international standard recommendations at the time of diagnosis [24]. All patients undergoing alloHCT had r/r disease following at least one therapy line. Both nodular and leukemic relapses were included. alloHCT was offered to patients meeting standard eligibility criteria, including adequate performance status (ECOG 0‐1), and hematopoietic cell transplantation comorbidity (HCT‐CI) score < 3. All patients had given written consent to academic outcome analysis; the FC cohort patients were treated within the prospective German Low‐Grade Lymphoma Study Group dose‐reduced allogeneic HCT Phase II protocol for relapsed follicular/MCL (GLSG‐DR alloHCT) [25]. Patients in the FC cohort were transplanted during the initial years of the study period in the context of the GLSG‐DR alloHCT protocol. In contrast, patients in the FBC cohort were transplanted predominantly in subsequent years, when myeloablative conditioning (MAC) had become the local standard. Recipients and related or unrelated donors were typed at HLA‐loci A, B, C, DR, and DQ, and a match of at least 9/10 loci at HLA A, B, C, DR, and DQ was considered eligible for transplantation. Stem cell grafts were not manipulated. For conditioning, patients received either MAC administered as FBC‐12 or FBC‐8 or reduced‐intensity conditioning (RIC) with FC regimens. FBC conditioning therapy consisted of fludarabine 25 mg/m^2^ per day from Day −8 to −4 before transplantation, busulfan 4 mg/kg or 3.2 mg/kg per day from Day −6 to −4 (FBC‐12) or from Day −6 to −5 (FBC‐8) and cyclophosphamide 60 mg/kg on Day −3 and −2. FC conditioning therapy consisted of fludarabine 30 mg/m^2^ per day from Day −7 to −4 and cyclophosphamide 30 mg/kg on Day −4 and −3. For unrelated or mismatched grafts, antithymocyte globulin (Grafalon, Neovii Biotech GmBH, Gräfelfing, Germany) 10 mg/kg or thymoglobulin 2 mg/kg (Genzyme, Sanofi, Frankfurt, Germany) was administered intravenously from Day 3 to 1 before transplantation. Although FBC was classified as myeloablative at the time of treatment (2001–2017), busulfan 8 mg/kg would now be categorized as reduced intensity by contemporary criteria (threshold > 9.2 mg/kg). Calcineurin inhibitor (CNI)‐based GvHD prophylaxis started on Day 1 before transplantation and consisted of tacrolimus 2 mg or ciclosporin 200 mg intravenously for 16 h and mycophenolate mofetil (1 g twice daily until Day 28 after transplantation). Tapering of CNI was generally started around Day +100 in the absence of graft‐versus‐host disease (GvHD) and discontinued after 6–9 months if no GvHD occurred. Ciclosporin was the recommended CNI option for patients in the FC cohort according to the GLSG‐DR alloHCT trial protocol.

## 3 Study and Statistics

The patients were divided into groups according to the following characteristics: age at diagnosis, sex, remission status, and disease control before transplantation, number of prior therapies, early or late relapse before transplantation, chemosensitivity of the disease, conditioning therapy, and donor grafts matched related (MRD), matched unrelated (MUD), and mismatch unrelated (mMUD). Acute and chronic GvHD were assessed, classified, and graded according to international standards [26, 27]. OS was defined as time from alloHCT to death from any cause. Progression‐free survival (PFS) was defined as time from alloHCT to relapse or progression or death from any cause. Kaplan–Meier estimates for PFS and OS were calculated from the time of alloHCT. NRM was defined as death without evidence of relapse or progression. NRM100 was defined as NRM by Day +100 after transplantation. Relapse was defined as evidence of MCL recurrence confirmed by histology, flow cytometry, or clinical progression. In addition, imaging with positron emission tomography/computed tomography (PET/CT) or standalone CT consistent with active disease were considered sufficient for relapse definition in patients without available biopsy. We analyzed the cumulative incidence of acute and chronic GvHD, NRM, and relapse. Late relapse was defined as recurrence of MCL beyond 12 months post‐alloHCT. Primary endpoints were defined as OS and PFS following alloHCT. Secondary endpoints included the relapse rate and time to relapse, subsequent treatment, and the incidence of acute and chronic GvHD as well as NRM. Multiple variables were analyzed regarding their impact on OS and PFS in univariate analysis using the log‐rank test. *p*‐values less than 0.05 were considered statistically significant. Variables with significant *p*‐values by univariate testing were subsequently included multivariate analysis using Cox proportional hazard ratios (HRs) with 95% confidence intervals (CIs). Median follow‐up was estimated using the reverse Kaplan–Meier method. Cumulative incidences of NRM and relapse were calculated using competing risk methodology (Gray's test), with death and relapse as competing events, respectively. Statistics and graphs were generated by IBM SPSS Statistics for Windows, Version 28.0, Armonk, NY: IBM Corp, and GraphPad Prism Version 9 for Windows, GraphPad Software, San Diego, California, USA, and in the R environment (v3.6.2) using the packages survival_3.4‐0 for survival analysis and swimplot v1.2.0, survminer v0.4.9, ggplot2 v3.3.6, and ggpubr 0.4.0 for graphical representation.

## 4 Results

### 4.1 Patient Characteristics and Treatment

Between 2001 and 2017, 29 patients with r/r MCL underwent alloHCT (Table 1), that is, before the approval of CAR‐T therapy for MCL (2020 in the United States; 2021 in Europe). The median time from diagnosis to transplant was 29 months. At the time of alloHCT, 69% of all patients (20/29) had chemosensitive disease (CR/PR) while the remaining patients (31%; 9/29) had SD/PD. The patients had received a median number of two treatment lines before alloHCT (range, 1–5), and 59% of patients had received high‐dose chemotherapy with autologous stem cell therapy (HDCT/ASCT) prior to alloHCT. In addition, three patients (10%) had received treatment with a covalent BTK inhibitor (ibrutinib) before alloHCT. Donor and graft characteristics are presented in Table 1. All patients had received peripheral blood stem cell (PBSC) grafts. There was no significant difference in the comparison of donors between the FBC and FC cohorts (*p =* 0.40). For GvHD‐prophylaxis, the patients had received predominantly tacrolimus in the FBC cohort and cyclosporine in the FC cohort, respectively. Otherwise, the cohorts were comparable regarding baseline characteristics (Table 1).

**TABLE 1:** Characteristics of the 29 MCL patients undergoing salvage alloHCT with FBC and FC.

Pts. at diagnosis, *n* (%)	All pts. (*n* = 29)	FBC 8/12 (*n* = 21)	FC (*n* = 8)	*p*‐value
Median age, years (range)	53 (37–67)	55 (37–67)	52 (44–63)	0.66
Gender (M/F)	21 (72)/8 (28)	17 (81)/4 (19)	4 (50)/4 (50)	0.10
Ann Arbor stage (available for 20 patients in FBC and 7 in FC):				
I–II	0 (0)	0 (0)	0 (0)	—
III	2 (7)	2 (10)	0 (0)	—
IV	25 (93)	18 (90)	7 (100)	0.40
MIPI score (available only for 13 patients in FBC):				
Low risk	—	4 (31)	—	—
Intermediate risk	—	3 (23)	—	—
High risk	—	6 (46)	—	—
Histology of Mantle cell lymphoma (available for eight in FBC/FC patients): 0.15				
Classic	9 (56)	3 (38)	6 (75)	—
Blastoid	7 (44)	5 (62)	2 (25)	—
Pts. at alloHCT *n* (%)	All pts.	FBC 8/12	FC	*p*‐value
Prior autoHCT: 0.41				
Yes	17 (59)	11 (52)	6 (75)	—
No	12 (41)	10 (48)	2 (25)	—
Median age at allo‐SCT, years (range):	56 (37–68)	59 (37–68)	54 (47–65)	0.94
Age ≤ 65 years	25 (86)	17 (81)	8 (100)	—
Age > 65 years	4 (14)	4 (19)	0 (0)	
Median Tx line before allo‐SCT (range):	2 (1–5)	2 (1–4)	3 (1–5)	0.24
≤ 2 Tx line before allo‐SCT	19 (66)	15 (71)	4 (50)	—
> 2 Tx line before allo‐SCT	10 (34)	6 (29)	4 (50)	—
Time from diagnosis to allo‐SCT, months (range):	29 (4–177)	25 (4–87)	31 (16–177)	0.26
Time to allo‐SCT ≤12 months	6 (21)	6 (29)	0 (0)	—
Time to allo‐SCT >12 months	23 (79)	15 (71)	8 (100)	0.09
Disease status before allo‐SCT: 0.31				
CR	5 (17)	3 (14)	2 (25)	—
PR	15 (52)	13 (62)	2 (25)	—
SD	1 (3)	1 (5)	0 (0)	—
PD	8 (28)	4 (19)	4 (50)	—
Stem cell source: 0.40				
BM/PB	0 (0)/29 (100)	0 (0)/21 (100)	0 (0)/8 (100)	—
MRD	10 (34)	8 (38)	2 (25)	—
MUD/mMUD	17 (59)/2 (7)	12 (57)/1 (5)	5 (62)/1 (13)	—
GvHD prophylaxis: < 0.001				
TAC + MMF	18 (62)	18 (86)	0 (0)	< 0.001
CsA + MMF	11 (38)	3 (14)	8 (100)	< 0.001
Conditioning regime:				
FBC 8	16 (55)	16 (76)	—	—
FBC 12	5 (17)	5 (24)	—	—
FC	8 (28)	—	8 (100)	
w/wo ATG	22 (76)/7 (24)	15 (71)/6 (29)	7 (87)/1 (13)	0.38

Abbreviations: alloHCT: allogeneic stem cell transplantation; ATG: antithymoglobulin; autoHCT: autologous stem cell transplantation; BM: bone marrow; CR: complete remission; CsA: cyclosporine; F: female; FBC: fludarabine, busulfan, cyclophosphamide; FC: fludarabine, cyclophosphamide; Haplo: haploid donor; M: male; MIPI: Mantle Cell Lymphoma International Prognostic Index; MMF: mofetil mycophenolate; mMUD: mismatch unrelated donor; MRD: match related donor; MUD: match unrelated donor; PB: peripheral blood; PD: progressive disease; PR: partial remission; Pts.: patients; SD: stable disease; TAC: tacrolimus; Tx: transplantation; w/wo: with/without.

### 4.2 Overall and PFS

The outcomes of alloHCT in MCL patients are presented in Table 2. Median follow‐up after alloHCT was 143 months (95% CI: 104–NR), reflecting short observation and the limited sample size (*n* = 8) in the FC cohort (median 143 months; 95% CI: 104–NR) versus longer follow‐up in the FBC cohort (median 162 months; 95% CI: NR–NR), with individual cases followed for up to 220 months (> 18 years). The relapse/progression rate in posttransplantation follow‐up was 38% (11/29). The median time to relapse/progression was 3 months (range 0–78 months). Eight posttransplantation relapses occurred within the first year after alloHCT, whereas 3 out of 11 (27%) patients experienced a late relapse > 12 months posttransplant. The 3‐year PFS and OS were 34% (95% CI: 21%–57%) and 41% (95% CI: 27%–64%), accordingly. Uni‐ and multivariate analyses revealed no impact of age, gender, time from first diagnosis to transplantation (</≥ 1‐year), number of treatment lines (≤ 2 vs. > 2), remission status at alloHCT, or prior autologous SCT on PFS and OS in both groups (Tables S1–S4). Patients with CR/PR prior to alloHCT showed a trend toward superior 3‐year OS compared to those with SD/PD (50% [95% CI: 32%–78%] vs. 22% [95% CI: 7%–75%]; HR: 2.35, 95% CI: 0.95–5.81; *p* = 0.06). A similar nonsignificant trend was observed for 3‐year PFS (40% [95% CI: 23%–68%] vs. 22% [95% CI: 7%–75%]; *p* = 0.15) (Figure S1).

**TABLE 2:** Outcomes of salvage alloHCT in patients of the study with FBC and FC.

Patients, *n* (%)	All pts. (*n* = 29)	FBC (*n* = 21)	FC (*n* = 8)	*p*‐value
Median FU after allo‐SCT, months (reverse Kaplan–Meier, 95% CI)	143 (104–NR)	143 (104–NR)	162 (NR–NR)	0.98
aGvHD, 0 vs. Grad I–IV	14 (48)/15 (52)	8 (38)/13 (62)	6 (75)/2 (25)	0.29
Incidence of cGvHD	18 (62)	13 (62)	5 (63)	0.97
cGvHD, 0 vs. limited vs. extensive	11 (38)/12 (41)/6 (21)	8 (38)/10 (48)/3 (14)	3 (37)/2 (25)/3 (37)	0.46
Relapse/progression in posttransplant FU	11 (38)	8 (38)	3 (37)	0.57
Median time to relapse/progression after allo‐SCT, months (range)	3 (0–78)	3 (0–78)	8 (3–12)	0.56
Relapse treatment following alloHCT:				
Radiotherapy	—	2 (25)	—	—
Radiotherapy followed by BTK inhibitor	—	1 (12.5)	—	—
BTK inhibitor	—	1 (12.5)	—	—
BSC	—	4 (50)	—	—
DLI	—	—	2 (67)	—
mTOR inhibitor	—	—	1 (33)	—
Response rate following radiotherapy:				
CR	—	2/3 (66)	—	—
PR	—	1/3 (34)	—	—
Remission rate at last FU: 0.59				
CR	20 (69)	15 (71)	5 (67)	—
PR	1 (3.5)	1 (5)	—	—
SD	1 (3.5)	1 (5)	—	—
r/r disease	7 (24)	4 (19)	3 (37)	—
Survival status at last FU (Alive)	8 (28)	7 (33)	1 (13)	0.27
Overall and progression‐free survival:				
3‐year OS	12 (41)	11 (52)	1 (13)	0.936
3‐year PFS	10 (34)	9 (43)	1 (13)	0.880
NRM100	2 (7)	1 (5)	1 (13)	—
3‐year NRM (CIF)	6 (21)	5 (24)	1 (13)	0.610
3‐year RI	13 (45)	7 (33)	6 (75)	0.055
Mortality reasons: 0.62				
r/r disease	6 (21)	4 (19)	2 (25)	—
Non‐lymphoma reasons	15 (52)	10 (48)	5 (63)	—
Infection / Infections associated with GvHD	12 (41)/8 (28)	7 (33)/5 (24)	5 (63)/3 (37)	—
Other reasons^a^	3 (10)	3 (14)	—	—

Abbreviations: aGvHD: acute graft‐versus‐host disease; BSC: best supportive care; BTK: Bruton tyrosin kinase; cGvHD: chronic GvHD; CR: complete response; CIF: cumulative incidence function; DLI: donor lymphocyte infusion; FU: follow‐up; mTOR: mammalian target of rapamycin; NRM: non‐relapse mortality; PR: partial response; r/r disease: relapsed/refractory disease; RI: relapse incidence; and SD: stable disease.

^a^ One patient, chronic renal failure requiring dialysis; two patients, unknown reason.

**FIGURE 1:**
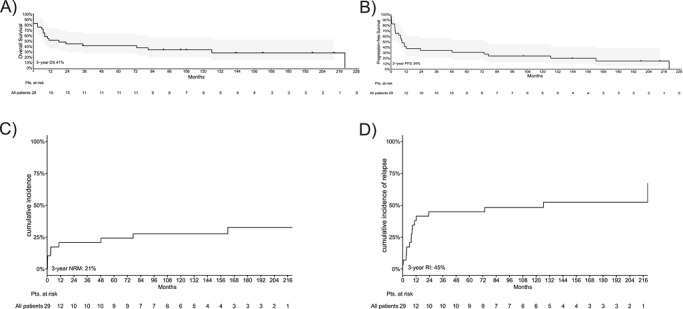
A 3‐year overall survival (OS), progression‐free survival (PFS), non‐relapse mortality (NRM), and relapse incidence (RI) of the entire cohort (*n* = 29) following allogeneic stem cell transplantation (alloHCT) in patients (pts) with mantle cell lymphoma (MCL). (A) OS, (B) PFS, (C) NRM, and (D) RI.

The estimated 3‐year PFS rates were 43% (95% CI: 26–70) in FBC and 13% (95% CI: 2–78) in FC patients (*p *= 0.880), the estimated 3‐year OS rates were respectively 52% (95% CI: 35–79) and 13% (95% CI:2–78), respectively (*p *= 0.936) (Figure 1 and Table 2).

Among eight patients who underwent alloHCT with progressive disease at the time of transplantation, outcomes were poor. In the FC cohort, four of eight patients had PD at transplant, all of whom experienced early progression, resulting in a 3‐year OS and PFS of 13%.

### 4.3 Follow‐Up, Outcome, and Subsequent Therapy

**FIGURE 2:**
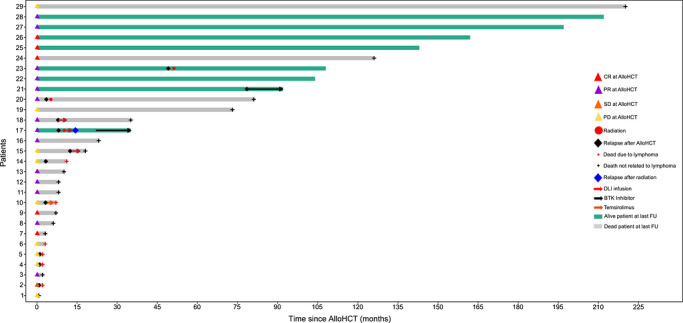
Swimmer plot showing time in months from allogeneic stem cell transplantation (alloHCT) to last follow‐up (FU) for each patient (*n* = 29). BTKi, Bruton tyrosine kinase inhibitor; CR, complete response; DLI, donor lymphocyte infusion; PD, progressive disease; PR, partial response; SD, stable disease.

Individual posttransplant disease trajectories, including relapse timing and salvage treatments, are visualized in Figure 2. Of the 11 patients with disease relapse, three patients with localized disease underwent radiation therapy, inducing a long‐term remission in two patients (#20 and #23; Figure 2). Of these, one patient died from an infection being in CR 81 months post‐alloHCT and 76 months post‐radiotherapy. The second patient (#23) was still alive and in CR at the last follow‐up 28 months after radiotherapy. One patient (#17) achieved CR following radiation, but relapsed 5 months thereafter, being alive and in CR at the last follow‐up following initiation of the non‐covalent BTK inhibitor pirtobrutinib. Two patients (#15 and #18) received DLI, but died from GvHD‐associated infections. One patient (#10) was treated with the mammalian target of rapamycin (mTOR) inhibitor everolimus and died due to disease progression (Figure 2).

### 4.4 GvHD, Relapse, and NRM

While the use of ATG or the availability of MRD donors were associated with significantly better OS in the whole cohort by univariate analysis (*p = *0.046 and 0.015) (Tables S1 and S2), the multivariate analysis revealed no significant predictor for outcome (Table S1).

Early NRM (NRM100) was 7% (2/29) with comparable rates in FC 13% (1/8) and FBC 5% (1/21). At 3 years, cumulative NRM was 24% (FC) (95% CI: 0%–37%) and 13% (95% CI: 5%–43%) (FBC). The 3‐year relapse incidence (RI) was 75% (FC) (95% CI: 38%–100%) and 33% (FBC) (95% CI: 12%–54%) (Figure S2). A landmark analysis excluding patients who died before Day +100 confirmed numerically better post‐NRM100 outcomes for FBC (3‐year PFS 76% [95% CI: 56%–100%] vs. 38% [95% CI: 8%–100%]; 3‐year OS 65% [95% CI: 46%–92%] vs. 20% [95% CI: 3%–100%]), but without statistical significance (*p* > 0.2).

Disease relapse and/or progression after alloHCT accounted for 21% (6/29) of deaths in the entire cohort. NRM was 21% (6/29) (95% CI: 6%–36%) at 3 years and 52% (15/29) (95% CI: 34–70) in the whole follow‐up. The 3‐year RI was 45% (13/29) (95% CI: 26%–64%). Overall, infection was the most common non‐lymphoma‐related cause of death, occurring in 41% (12/29) of patients in the entire follow‐up. Other causes of death (chronic renal failure requiring dialysis, *n* = 1; unknown reason, *n* = 2) accounted for 10% (3/29).

## 5 Discussion

AlloHCT has gradually shifted from a broadly applied strategy in r/r MCL to a selective option reserved for biologically high‐risk or treatment‐refractory patients in the modern era of targeted and cellular therapies [22, 28]. In this retrospective single‐center cohort with follow‐up extending beyond 15 years in individual cases, alloHCT achieved 3‐year PFS and OS of 34% and 41%, consistent with historical evidence and confirming the capacity of GvL activity to induce long‐term disease control in a subset of patients. Although the number of alloHCT procedures has declined, our data illustrate that durable remission, including treatment‐free survival beyond 10 years, remains achievable and is, based on current follow‐up, not yet reproducible with any other therapeutic modality in r/r MCL [29].

Conditioning intensity did not translate into statistically significant survival differences, but outcomes were numerically superior in patients receiving the FBC regimen compared with FC. In the contemporary treatment landscape, reduced‐intensity or non‐MAC represents the preferred approach in patients with r/r MCL. Our cohort reflects historical clinical practice over a prolonged study period, during which myeloablative regimens such as FBC were more frequently applied. Baseline characteristics were largely comparable between groups. However, a higher proportion of patients in the FC cohort had progressive disease at the time of alloHCT, which may have contributed to inferior outcomes. In addition, follow‐up duration differed substantially between cohorts, with shorter observation time in the FC group. These findings suggest that disease status at transplantation may have a greater impact on outcome than conditioning intensity itself. Patients transplanted with active progressive disease, particularly within the FC group, experienced uniformly poor outcomes, reinforcing the longstanding principle that disease control at the time of alloHCT remains one of the strongest clinical determinants of benefit [30]. In our cohort, this association did not reach statistical significance, most likely due to the limited sample size. However, a clear trend toward inferior outcomes in patients with PD/SD was observed, supporting the clinical relevance of disease control prior to transplantation. Early NRM (NRM100) was 7% in the entire cohort and 5% in the FBC subgroup. Although still relevant, these toxicity rates contrast with the lower treatment‐related mortality reported with current CAR‐T products. However, recent real‐world data indicate that NRM after CAR‐T for MCL exceeds 10% after a median follow‐up of 13.4 months, highlighting that toxicity‐related mortality remains a clinically relevant concern [14]. A recent EBMT‐based analysis using propensity score matching to compare CAR‐T cell therapy with alloHCT reported higher early NRM after alloHCT, but comparable long‐term OS between both approaches [31]. These findings support the notion that, despite increased early toxicity, alloHCT remains a potentially curative option with durable disease control in selected patients. CAR‐T eligibility, feasibility of cell collection, and response durability are not universal and continue to limit access or benefit for a relevant subset of patients [32].

A distinctive observation in this cohort was the relapse pattern. While the majority of relapses occurred within the first year after transplantation, approximately one‐quarter of relapses developed beyond 12 months and were predominantly localized. These late events were frequently salvageable with radiotherapy alone or combined with BTK inhibition and resulted in renewed durable remission in several cases. This behavior differs markedly from the systemic relapse patterns typically seen after targeted therapy or CAR‐T failure and suggests persistent systemic immune control with isolated immune escape, a phenomenon consistent with ongoing GvL activity [32]. Such observations highlight an important clinical nuance. alloHCT does not merely delay relapse, but in selected patients, can fundamentally alter the relapse biology and maintain curative potential even after recurrence.

Posttransplant salvage strategies in the present cohort included radiotherapy, DLI, mTOR inhibition, and both covalent and non‐covalent BTK inhibitor therapy. The latter is increasingly relevant, as modern sequencing studies suggest that BTKi retain activity posttransplant and can be reused following immune‐mediated disease control [13]. Early experience with CD20 × CD3 bispecific antibodies further expands potential salvage options and may complement or replace DLI in future practice [33]. While numbers remain small, these developments indicate that alloHCT does not terminate the therapeutic pathway, but rather preserves meaningful rescue strategies, an advantage not yet demonstrated after CAR‐T relapse.

The evolving treatment sequence in r/r MCL now frequently follows a BTKi‐to‐CAR‐T treatment sequence; however, 40%–60% of patients relapse within 3–5 years after CAR‐T, and the prognosis after CAR‐T failure remains poor [10, 32]. Several emerging analyses suggest that alloHCT after CAR‐T may still be feasible and effective, providing a potential consolidation strategy for high‐risk disease biology such as TP53 alterations, blastoid morphology, or rapid progression. Conversely, whether earlier referral for alloHCT prior to overt treatment exhaustion would improve long‐term outcomes remains unanswered [21, 28]. As precision medicine advances, the future decision point may no longer be if alloHCT is performed, but which patients should undergo alloHCT before losing the biological window in which immune‐mediated cure is still possible.

The present study has limitations inherent to its retrospective, single‐center nature and small sample size, particularly within the FC subgroup. Follow‐up was heterogeneous, and key biological risk variables, including TP53 status, Ki‐67, and MIPI, were not uniformly available, preventing detailed molecularly stratified analysis. The standard Kaplan–Meier and cumulative incidence functions were used, which may modestly overestimate relapse probability in the presence of early NRM. GvHD prophylaxis differed between cohorts (tacrolimus/MMF in FBC vs. cyclosporine/MMF in FC), which may have contributed to differences in GvHD rates and NRM. Supportive care practices, conditioning classification, and transplant selection evolved over the 16‐year study period, further limiting direct comparability with contemporary cohorts. Nevertheless, the depth of clinical annotation, long observation period, and documentation of individual salvage trajectories provide insights unavailable from registry‐level datasets or modern cellular therapy trials with shorter follow‐up. To refine the future role of alloHCT in relation to BTKi, CAR‐T, and bispecific antibodies, larger multicenter datasets with harmonized long‐term follow‐up will be required. Prospective registry infrastructures will therefore be essential to define which patients still benefit from alloHCT in the modern therapeutic era.

Taken together, these findings argue against the assumption that alloHCT has been rendered obsolete. Instead, its role has become narrower but more strategic. It remains the only modality with proven curative potential outside clinical trial settings, capable of generating treatment‐free survival and enabling salvage after relapse. In an era where CAR‐T therapy is transformative but not definitively curative, and where post‐CAR‐T relapse remains a critical unmet need, alloHCT may serve as an essential option in selected patients, particularly those with primary BTKi failure, high‐risk biological features, or relapse after CAR‐T.

## 6 Conclusion

AlloHCT remains a valid and potentially curative strategy for biologically high‐risk or post‐CAR‐T relapsed MCL, particularly when applied with adequate disease control at transplant. Its relevance will persist alongside not in competition with targeted and cellular therapies, provided patient selection and sequencing are guided by biology rather than chronology.

## Author Contributions

Conceptualization: Enver Aydilek, Evgenii Shumilov, Justin Hasenkamp, and Gerald Wulf. Data curation: Enver Aydilek, Markus Maulhardt, Evgenii Shumilov, and Tobias Tix. Investigation: Enver Aydilek, Justin Hasenkamp, Evgenii Shumilov, and Gerald Wulf. Methodology: Enver Aydilek, Justin Hasenkamp, Evgenii Shumilov, and Gerald Wulf. Supervision: Evgenii Shumilov, Justin Hasenkamp, and Gerald Wulf. Validation: Enver Aydilek, Justin Hasenkamp, Paolo Mazzeo, Tobias Tix, Evgenii Shumilov, and Gerald Wulf. Visualization: Enver Aydilek, Evgenii Shumilov, and Paolo Mazzeo. Writing – original draft: Enver Aydilek and Evgenii Shumilov. Writing – review and editing: Enver Aydilek, Justin Hasenkamp, Tobias Tix, Evgenii Shumilov, and Gerald Wulf. All authors have read and agreed to the published version of the manuscript.

## Funding

The authors have nothing to report.

## Ethics Statement

The study was conducted according to the guidelines of the Declaration of Helsinki and approved by the Ethics Committee of the University Medical Center Göttingen No. 6/3/23.

## Consent

The authors have nothing to report.

## Conflicts of Interest

Enver Aydilek has received honoraria/consultancy: Kite/Gilead, AstraZeneca, J&J, BMS, Takeda, Abbvie, and Lilly; travel/meeting support: J&J and AstraZeneca; advisory boards: AstraZeneca, Kite/Gilead, J&J, and Pfizer. The other authors declare no conflicts of interest.

## Supporting information




**Supporting file 1**: jha270318‐sup‐0001‐tableS1‐S4.docx

**Supporting file 2**: jha270318‐sup‐0002‐figureS1.pdf

**Supporting file 3**: jha270318‐sup‐0003‐figureS2.pdf

## Data Availability

The data that support the findings of this study are not publicly available due to privacy reasons, but can be obtained from the corresponding author upon reasonable request.
