# Supplementary material for: Allogeneic Hematopoietic Cell Transplantation for Relapsed or Refractory Mantle Cell Lymphoma: Real‐World Outcomes, Late Relapse Patterns, and Clinical Utility in the Chimeric Antigen Receptor T‐Cell Era
Source: EJHaem. 2026 May 26;7(3):e70318. doi: 10.1002/jha2.70318 (PMC13240352; doi:10.1002/jha2.70318)
Supplement: Supplementary file 1 — Supporting file 1: jha270318‐sup‐0001‐tableS1‐S4.docx [file JHA2-7-e70318-s003.docx]

**Supplemental Table 1:** Univariate and multivariate analysis of OS in all patients (n=29)

| Variable | | Univariate p |  | Multivariate p | | 95% CI | | |  |  |
| --- | --- | --- | --- | --- | --- | --- | --- | --- | --- | --- |
|  |  | |  | |  | Lower limit | | Upper limit | | |
| *Gender M/F* | | 0.743 | n.s. | |  |  | |  | | |
| *MIPI at diag.* | | 0.892 | n.s. | |  |  | |  | | |
| *Ann-Arbor St. III vs. IV at diag.* | | 0.569 | n.s. | |  |  | |  | | |
| *B-symptomatic at diag.* | | 0.806 | n.s. | |  |  | |  | | |
| *Age at relapse ≤/> 65 years* | | 0.624 | n.s. | |  |  | |  | | |
| *Early relapse y/n* | | 0.272 | n.s. | |  |  | |  | | |
| *Relapse before alloSCT y/n* | | 0.476 | n.s. | |  |  | |  | | |
| *Prior autoSCT y/n* | | 0.852 | n.s. | |  |  | |  | | |
| *Lines chemotherapy ≤/> 2* | | 0.770 | n.s. | |  |  | |  | | |
| *Age at alloSCT*  *≤/> 65 years* | | 0.202 | n.s. | |  |  | |  | | |
| *Time Diag. to alloSCT ≤/> 12 m* | | 0.616 | n.s. | |  |  | |  | | |
| *PD vs. CR, SD, PR* | | 0.152 | n.s. | |  |  | |  | | |
| *MRD / MUD, mMUD* | | 0.302 | n.s. | |  |  | |  | | |
| *MRD-no-ATG vs. MUD, MMD+ATG* | | 0.015 | * | | 0.117 | 0.139 | | 1.246 | | |
| *FBC-8 vs. FBC-12* | | 0.600 | n.s. | |  |  | |  | | |
| *ATG vs. no ATG* | | 0.046 | * | | 0.014 | 1.515 | | 41.149 | | |
| *aGvHD y/n* | | 0.214 | n.s. | |  |  | |  | | |
| *Relapse after allo y/n* | | 0.931 | n.s. | |  |  |  | | |  |

M (male), F (female), n.s. (not significant), CI (confidence interval), MIPI (Mantle cell lymphoma interanational prognotstic index), diag. (diagnosis), St. (Stage), y (yes), n (no), alloSCT (allogeniec stem cell transplantation), PD (progressive disease), CR (complete remission), SD (stable disease), PR (partial response), MRD (match related donor), MUD (match unrelated donor), mMUD (mismatch unrelated donor), MMD (mismatch donor) ATG (antithymoglobulin), FBC-8/12 (combination of fludarabine, busulfan at 8/12 mg/kg body weight and 120 mg/kg cyclophosphamide), aGvHD (acute graft-versus-host disease).

**Supplemental Table 2:** Univariate and multivariate analysis of PFS in all patients (n=29)

| Variable | | Univariate p |  | Multivariate p | | 95% CI | | |  |  |
| --- | --- | --- | --- | --- | --- | --- | --- | --- | --- | --- |
|  |  | |  | |  | Lower limit | | Upper limit | | |
| *Gender M/F* | | 0.468 | n.s. | |  |  | |  | | |
| *MIPI at diag.* | | 0.986 | n.s. | |  |  | |  | | |
| *Ann-Arbor St. III vs. IV at diag.* | | 0.873 | n.s. | |  |  | |  | | |
| *B-symptomatic at diag.* | | 0.570 | n.s. | |  |  | |  | | |
| *Age at relapse ≤/> 65 years* | | 0.261 | n.s. | |  |  | |  | | |
| *Early relapse y/n* | | 0.193 | n.s. | |  |  | |  | | |
| *Relapse before alloHCT y/n* | | 0.160 | n.s. | |  |  | |  | | |
| *Prior autoHCT y/n* | | 0.768 | n.s. | |  |  | |  | | |
| *Lines chemotherapy ≤/> 2* | | 0.959 | n.s. | |  |  | |  | | |
| *Age at alloHCT*  *≤/> 65 years* | | 0.177 | n.s. | |  |  | |  | | |
| *Time Diag. to alloHCT ≤/> 12 m* | | 0.969 | n.s. | |  |  | |  | | |
| *PD vs. CR, SD, PR* | | 0.345 | n.s. | |  |  | |  | | |
| *MRD / MUD, mMUD* | | 0.064 | n.s. | |  |  | |  | | |
| *MRD-no-ATG vs. MUD, MMD+ATG* | | 0.127 | n.s. | |  |  | |  | | |
| *FBC-8 vs. FBC-12* | | 0.734 | n.s. | |  |  | |  | | |
| *ATG vs. no ATG* | | 0.127 | n.s. | |  |  | |  | | |
| *aGvHD y/n* | | 0.253 | n.s. | |  |  | |  | | |
|  |  |  |  | |  |  |  | | |  |

M (male), F (female), n.s. (not significant), CI (confidence interval), MIPI (Mantle cell lymphoma international prognostic index), diag. (diagnosis), St. (Stage), y (yes), n (no), alloHCT (allogeneic stem cell transplantation), PD (progressive disease), CR (complete remission), SD (stable disease), PR (partial response), MRD (match related donor), MUD (match unrelated donor), mMUD (mismatch unrelated donor), ATG (antithymoglobulin), FBC-8/12 (combination of fludarabine, busulfan at 8/12 mg/kg body weight and 120 mg/kg cyclophosphamide), aGvHD (acute graft-versus-host disease).

**Supplemental Table 3:** Univariate and multivariate analysis of OS in the FBC cohort.

| Variable | | Univariate p |  | Multivariate p | | 95% CI | | |  |  |
| --- | --- | --- | --- | --- | --- | --- | --- | --- | --- | --- |
|  |  | |  | |  | Lower limit | | Upper limit | | |
| *Gender M/F* | | 0.861 | n.s. | |  |  | |  | | |
| *MIPI at diag.* | | 0.892 | n.s. | |  |  | |  | | |
| *Ann-Arbor St. III vs. IV at diag.* | | 0.676 | n.s. | |  |  | |  | | |
| *B-symptomatic at diag.* | | 0.890 | n.s. | |  |  | |  | | |
| *Age at relapse^1^ ≤/> 65 years* | | - | - | |  |  | |  | | |
| *Early relapse y/n* | | 0.343 | n.s. | |  |  | |  | | |
| *Relapse before alloHCT y/n* | | 0.626 | n.s. | |  |  | |  | | |
| *Prior autoHCT y/n* | | 0.516 | n.s. | |  |  | |  | | |
| *Lines chemotherapy ≤/> 2* | | 0.718 | n.s. | |  |  | |  | | |
| *Age at alloHCT*  *≤/> 65 years* | | 0.424 | n.s. | |  |  | |  | | |
| *Time Diag. to alloHCT ≤/> 12 m* | | 0.425 | n.s. | |  |  | |  | | |
| *PD vs. CR, SD, PR* | | 0.688 | n.s. | |  |  | |  | | |
| *MRD / MUD, mMUD* | | 0.152 | n.s. | |  |  | |  | | |
| *MRD-no-ATG vs. MUD, MMD+ATG* | | 0.025 | * | | 0.060 | 0.922 | | 56.633 | | |
| *FBC-8 vs. FBC-12* | | 0.600 | n.s. | |  |  | |  | | |
| *ATG vs. no ATG* | | 0.025 | * | | - - - - - ^2^ | - - - - - | | - - - - - | | |
| *aGvHD y/n* | | 0.204 | n.s. | |  |  | |  | | |
| *Relapse after allo y/n* | | 0.605 | n.s. | |  |  |  | | |  |

M (male), F (female), n.s. (not significant), CI (confidence interval), MIPI (Mantle cell lymphoma international prognostic index), diag. (diagnosis), St. (Stage), y (yes), n (no), alloHCT (allogeneic stem cell transplantation), PD (progressive disease), CR (complete remission), SD (stable disease), PR (partial response), MRD (match related donor), MUD (match unrelated donor), mMUD (mismatch unrelated donor), ATG (antithymoglobulin), FBC-8/12 (combination of fludarabine, busulfan at 8/12 mg/kg body weight and 120 mg/kg cyclophosphamide), aGvHD (acute graft-versus-host disease).

^1^ An analysis could not be conducted as there was only one patient >65 years.

^2^ Not feasible for the multivariate analysis due to linearly dependent covariant.

**Supplemental Table 4:** Univariate and multivariate analysis of PFS in the FBC cohort.

| Variable | | Univariate p |  | Multivariate p | | 95% CI | | |  |  |
| --- | --- | --- | --- | --- | --- | --- | --- | --- | --- | --- |
|  |  | |  | |  | Lower limit | | Upper limit | | |
| *Gender M/F* | | 0.557 | n.s. | |  |  | |  | | |
| *MIPI at diag.* | | 0.986 | n.s. | |  |  | |  | | |
| *Ann-Arbor St. III vs. IV at diag.* | | 0.847 | n.s. | |  |  | |  | | |
| *B-symptomatic at diag.* | | 0.542 | n.s. | |  |  | |  | | |
| *Age at relapse^1^ ≤/> 65 years* | | - | - | |  |  | |  | | |
| *Early relapse y/n* | | 0.636 | n.s. | |  |  | |  | | |
| *Relapse before alloHCT y/n* | | 0.161 | n.s. | |  |  | |  | | |
| *Prior autoHCT y/n* | | 0.653 | n.s. | |  |  | |  | | |
| *Lines chemotherapy ≤/> 2* | | 0.521 | n.s. | |  |  | |  | | |
| *Age at alloHCT*  *≤/> 65 years* | | 0.153 | n.s. | |  |  | |  | | |
| *Time Diag. to alloHCT ≤/> 12 m* | | 0.823 | n.s. | |  |  | |  | | |
| *PD vs. CR, SD, PR* | | 0.572 | n.s. | |  |  | |  | | |
| *MRD / MUD, mMUD* | | 0.065 | n.s. | |  |  | |  | | |
| *MRD-no-ATG vs. MUD, MMD+ATG* | | 0.121 | n.s. | |  |  | |  | | |
| *FBC-8 vs. FBC-12* | | 0.734 | n.s. | |  |  | |  | | |
| *ATG vs. no ATG* | | 0.121 | n.s. | | - - - - - ^2^ | - - - - - | | - - - - - | | |
| *aGvHD y/n* | | 0.255 | n.s. | |  |  | |  | | |
|  |  |  |  | |  |  |  | | |  |

M (male), F (female), n.s. (not significant), CI (confidence interval), MIPI (Mantle cell lymphoma international prognostic index), diag. (diagnosis), St. (Stage), y (yes), n (no), alloHCT (allogeneic stem cell transplantation), PD (progressive disease), CR (complete remission), SD (stable disease), PR (partial response), MRD (match related donor), MUD (match unrelated donor), mMUD (mismatch unrelated donor), ATG (antithymoglobulin), FBC-8/12 (combination of fludarabine, busulfan at 8/12 mg/kg body weight and 120 mg/kg cyclophosphamide), aGvHD (acute graft-versus-host disease).

^1^ An analysis could not be conducted as there was only one patient >65 years.

^2^ Not feasible for the multivariate analysisdue to linearly dependent covariant.
