# Supplementary figures and images for: Allogeneic Hematopoietic Cell Transplantation for Relapsed or Refractory Mantle Cell Lymphoma: Real‐World Outcomes, Late Relapse Patterns, and Clinical Utility in the Chimeric Antigen Receptor T‐Cell Era
Source: EJHaem. 2026 May 26;7(3):e70318. doi: 10.1002/jha2.70318 (PMC13240352; doi:10.1002/jha2.70318)

A)

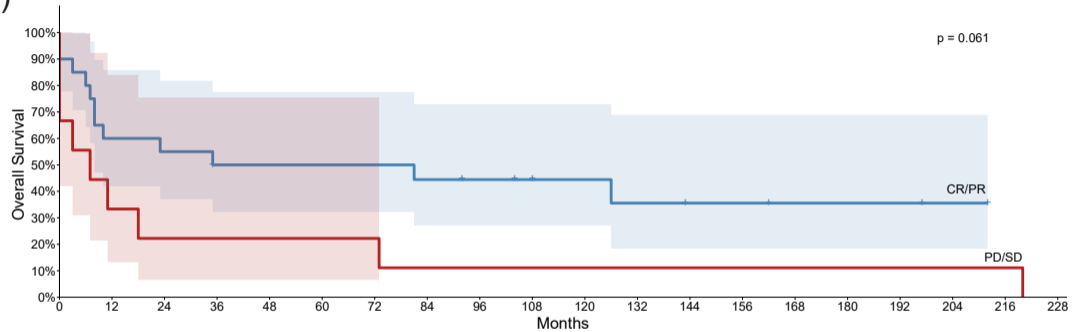[illegible]

B)

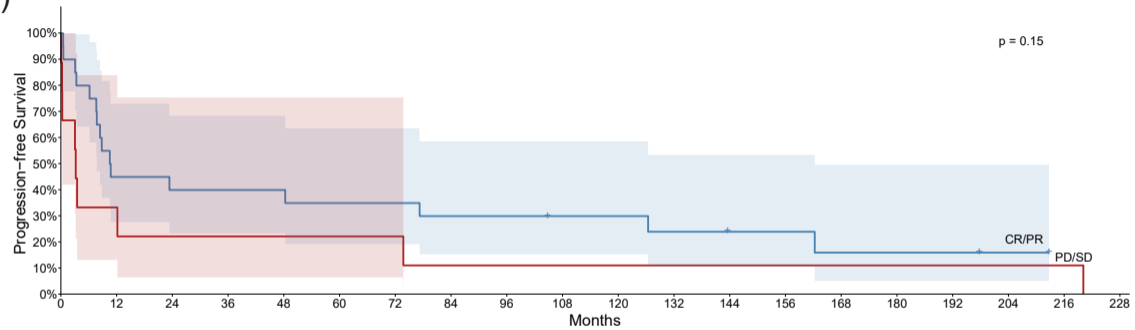[illegible]

Supplement: Supplementary file 2 — Supporting file 2: jha270318‐sup‐0002‐figureS1.pdf [file JHA2-7-e70318-s001.pdf]

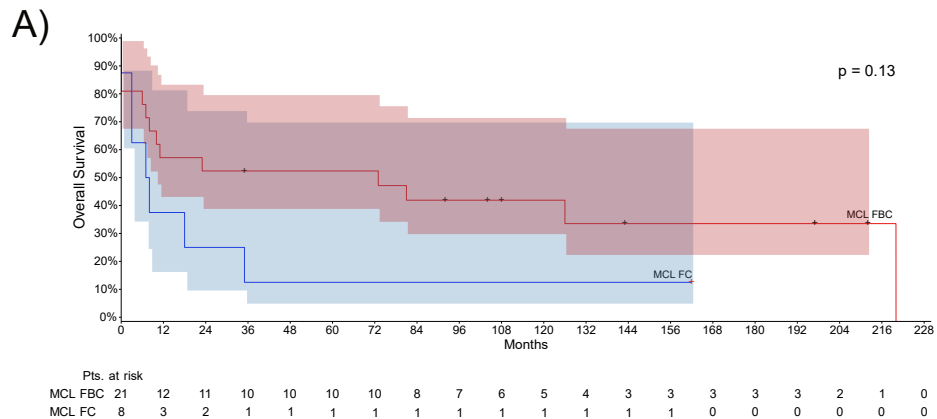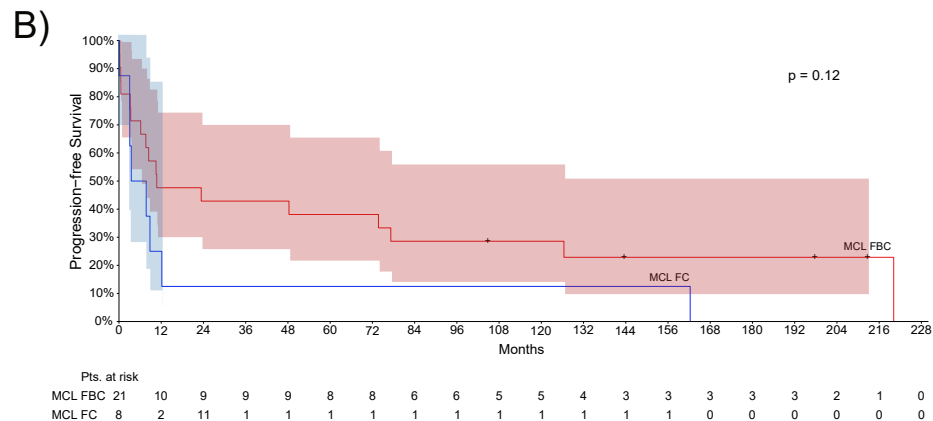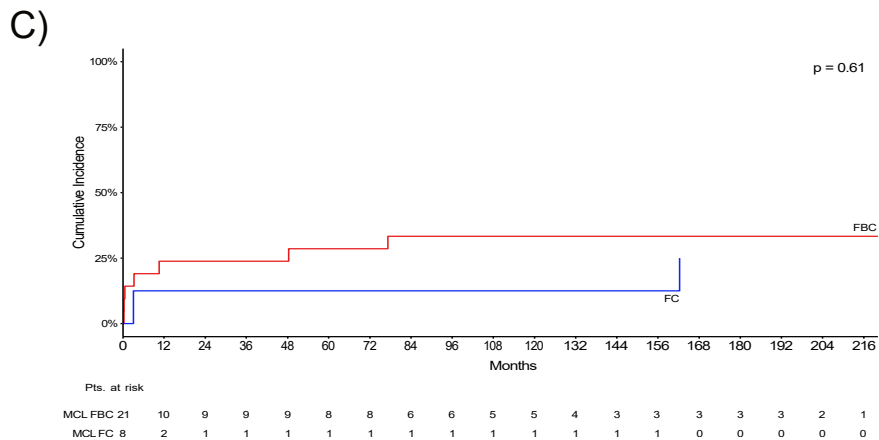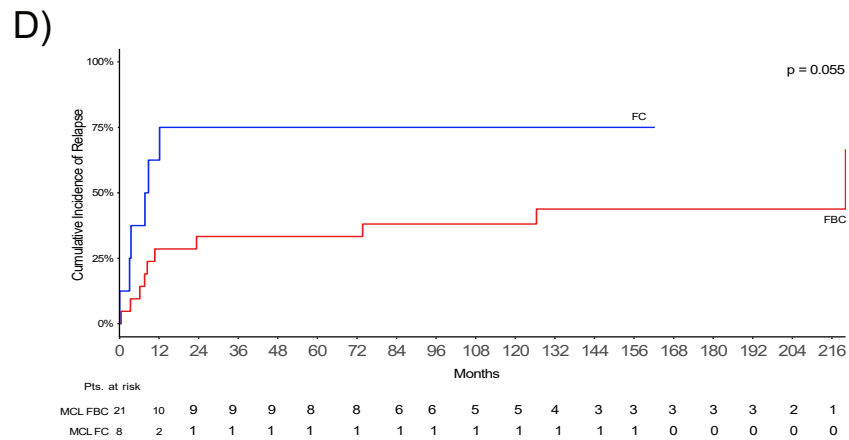

Supplement: Supplementary file 3 — Supporting file 3: jha270318‐sup‐0003‐figureS2.pdf [file JHA2-7-e70318-s002.pdf]
